# Data-driven decisions: a practical toolkit for librarians and information professionals

**DOI:** 10.29173/jchla29751

**Published:** 2024-04-01

**Authors:** Pamela S. Morgan

**Affiliations:** Former Division Head, Information Resources and Archives, Health Sciences Library, Memorial University of Newfoundland St. John’s, NL, Canada

Stubbing A. **Data-driven decisions: a practical toolkit for librarians and information professionals**. London, UK: Facet Publishing, 2022. Paperback: 200 p. ISBN: 978-1-7833-0478-3. Price: USD$71.00. Available from: https://www.alastore.alaorg/datadriven

Show value, prove your worth, get bang for your buck, do more with less, demonstrate impact. Regardless of the phrasing, the underlying concept of evidence-based practice is one that all libraries are familiar with, whether you are working solo in a hospital library or in a large academic health library with many colleagues. Evidence-based practice is ingrained in all aspects of health librarianship, from evaluating a service, to making collections decisions, to even justifying your very existence. And evidence requires data, whether quantitative data or qualitative data. Unfortunately, many librarians may feel ill-equipped to deal with data, all the more if they do not have a colleague to lead the way, or if they have a variety of responsibilities such that they just do not have the time and energy to do a deep dive into learning about assessment. This little book hopes to change all that by making data collection and analysis easy and accessible.

The main author, Amy Stubbing, is the Academic Engagement Lead at the University of Westminster. She has been focused on using data, making data-driven decisions, and promoting data literacy throughout her library career. Her belief that there is a gap in data literacy training and resources for librarians has led to this book, with the aim of providing librarians with a level of knowledge so that they feel comfortable with data collection, analysis, reporting, and decision making.

Because it is written for librarians who are not confident with data, Stubbing steps through all the basics, making no assumptions that the reader has any serious knowledge or skillset when it comes to data. But she doesn’t talk down to you either. Indeed, the book reads like a casual chat with a mentor. It has a very informal tone, the chapters are relatively short, and the writing is very easy to digest. The numerous examples are clear and readily relatable for any librarian in any type of library.

The layout of each chapter follows a pattern, with a chapter summary and a chapter table of contents that allows for diving into specific areas. There are often cross references within chapters to refer to specific sections in the toolkit. There are “tip” boxes to highlight specific concepts, the tables are useful and detailed, and the figures demonstrate concepts being described.

In the introduction, the author uses her own experience to explain why she wrote the book and why you should read it, starting with how she got into data analysis and how she used evidence culled from data to change services. The book then splits into two distinct sections. Part one is a step-by-step toolkit written entirely by the author. It is comprised of six steps: identify, collect, map, analyse, act, and review. Part two contains reflections by several contributors on putting the concepts in the toolkit into practice in a library.

One of the keys emphasized in the toolkit is that you absolutely must have a purpose for your data, and not simply collect data for the sake of collecting data. You must start with what you are trying to achieve, break your need into specific questions, and then determine what data will answer the questions. The toolkit does not just describe what you should do, but identifies pitfalls and bad habits, and explains why you do specific things in a specific order. Also included in the toolkit are tips on reporting your data and communicating your decisions.

Another key point is that planning your data collection is critical to be able to manipulate the data to get it into a structure whereby you can use it do your analysis and get your evidence. One example of how thorough and clear the text is, is that the author explains the importance of storing and organizing the data the right way, illustrates this with an example of a poor spreadsheet, explains the issues, and then shows a spreadsheet with the same data reformatted in a way that is better suited to answering the question.

I found the author’s distinction between data mapping and data analysis to be insightful, as I have always lumped them into one step. She sees data mapping as the step between collection and analysis; the translating, coding, visualising, and normalizing of data to enable comparisons of data from different sources.

Part two contains detailed descriptions of what you can do with your data and how to use the toolkit for specific data tasks. These include a chapter on managing staff, using data to illustrate what your team is doing, instead of just stating “we’re busy”; a collections chapter on using data to understand your collection; a user experiences (UX) chapter that includes a summary look at techniques for doing UX research; and a chapter on alternative data such as social media, google analytics, website hits, and gate or head counts. The book culminates in two expansive case studies. The chapter on building a data culture was particularly excellent, including examples of how poor data spreadsheets impacted the project, and insight into analysis, engagement, and reporting.

The author wants to “give you a solid foundation on your data journey” and I believe she has accomplished this goal. She uses real life examples from libraries making it easy to see how the information is relevant to your library. I have over 30 years in academic health libraries, working frequently with collections data for usage analysis and decision making about purchases, renewals, and cancellations. Collections was where I developed my own data skills, and I learned pretty much all of it on the job. This would have been a great book to have starting out to learn about data collection and analysis in a more systematic way without all the backtracking and missteps. If you are just starting out with data analysis or developing an evidence-based research project, or if you have always wanted to but were never sure where to get started, or if you are in need of a data mentor, then this book is for you. I recommend it to anyone who needs to build their confidence and skills in applying evidence-based practice within their own library.

